# Consumption of Dairy Products and the Risk of Developing Breast Cancer in Polish Women

**DOI:** 10.3390/nu13124420

**Published:** 2021-12-10

**Authors:** Bożena Wajszczyk, Jadwiga Charzewska, Dariusz Godlewski, Brunon Zemła, Elżbieta Nowakowska, Maciej Kozaczka, Małgorzata Chilimoniuk, Dorothy R. Pathak

**Affiliations:** 1 Department of Nutrition and Nutritional Value of Food, National Institute of Public Health NIH-National Research Institute, 00-791 Warszawa, Poland; 2 Center of Cancer Prevention and Epidemiology OPEN, 61-863 Poznań, Poland; godlewski.open@wp.pl; 3 Institute of Oncology, 44-102 Gliwice, Poland; Brunon.Zemla@io.gliwice.pl; 4 Wielkopolska Center of Oncology, 61-866 Poznań, Poland; elzbieta.nowakowska@affidea.com; 5 II Clinic of Radiology and Chemiotherapy, National Institute of Oncology, 44-102 Gliwice, Poland; kozaczkam@interia.pl; 6 Bialystok Center of Oncology, 15-027 Bialystok, Poland; mchilimoniuk@vp.pl; 7 Department of Epidemiology and Biostatistics, Michigan State University, East Lansing, MI 48824, USA

**Keywords:** breast cancer, milk, dairy products, menopausal status

## Abstract

Lack of consistency in the relationship between dairy products consumption and breast cancer (BC) risk motivated us to evaluate this association in a case-control study of BC among Polish women. The study includes 1699 women 26–79 years of age, 823 BC cases identified in Cancer Registries and 876 randomly selected controls from the national population registry. Using a validated, semiquantitative food frequency questionnaire (FFQ), the consumption of dairy products was collected for a time period of 10–15 years prior to BC diagnosis. We used logistic regression, adjusting for potential confounders, to assess the relationship between total dairy consumption as well as individual dairy groups of milk, cottage cheese and hard cheese and BC risk for premenopausal and postmenopausal women. For total consumption, a significant decrease in BC risk was observed with increased consumption of one serving/week, OR _trend_ = 0.98, 2% decrease in risk, for premenopausal women only. For milk, a significant decrease in BC risk was observed for an increase in consumption of one glass/week, OR _trend_ = 0.95, 5% decrease, in both strata of menopause. In contrast, for hard cheese, a significant increase in the risk of 10% was observed only in premenopausal women, OR _trend_ = 1.10. Cottage cheese consumption significantly reduced BC risk by 20%, OR _trend_ = 0.80, for an increase in one serving/week for postmenopausal women only. Our results show that individual dairy products have a statistically significant but bi-directional relationship with BC risk, which differs for premenopausal and postmenopausal women.

## 1. Introduction

The relationship between the consumption of selected food groups, individual foods or their nutrients and risk of various cancers has been extensively studied globally, with inconsistent results. Wiseman (2018) [1], based on the evidence gathered by the Continuous Update Project (CUP) of WCRF/AICR 3rd Expert Report Diet, Nutrition, Physical Activity and Cancer: a Global Perspective (2018) [2], provided a summary of specific lifestyle and dietary exposures, reviewed by an independent panel of experts, for which there was strong evidence (defined as “convincingly ”or“ probably” causal) for an increase or decrease in risk of one or more cancers. The group of dairy products and the calcium they contain were considered as having evidence for a strong causal relationship of reduced risk only in the development of colorectal cancer.

The assessment of the relationship between breast cancer risk and the group of dairy products has been inconsistent, as observed in several meta-analysis publications. Moorman and Terry, in their 2004 review of over 40 studies, pointed out the variations in the ways that dairy products consumption are analyzed and reported. Some studies provide information for total dairy products consumption, while others break it down into components such as milk products, cheeses, or fermented milk, while still others group them into categories of low-fat products vs. high-fat products. The timing of consumption is also considered in some studies, such as childhood vs. adulthood. Among the studies reviewed, the effect of total dairy consumption on breast cancer risk was evaluated in three cohorts and nine case-control studies. The three cohorts observed a significant inverse relationship between breast cancer risk and total dairy products consumption, with one study observing it only for premenopausal women. The results for the nine case-control studies were highly inconsistent: two observed inverse association, five no association and two observed increased breast cancer risk. By summarizing the results of over 40 studies reviewed, which evaluated the effect of total dairy intake as well as the various subgroups of dairy products on breast cancer risk, they concluded that consumption of dairy products did not show a consistent relationship with the risk of breast cancer [3]. The most recent meta-analysis of 36 studies, evaluating total consumption, as well as fermented vs. non-fermented and high-fat vs. low-fat dairy products, concluded that total dairy products have a protective effect on women in general (Hazard Ratio = 0.95, 95% CI = 0.91–0.99), especially when evaluated by estrogen/progesterone receptor status [4]. The study also concludes that fermented dairy was shown to reduce BC risk in postmenopausal women and low-fat dairy in premenopausal women. The publication draws attention to the observation that different dairy products may have a different effect on the tested final effect. A meta-analysis of 18 prospective cohort studies [5] reported a significant reduction in relative risk for the highest vs. lowest total dairy foods consumption. An analysis of dose–response (total dairy intake available from eight cohort studies, milk from nine) observed a slight reduction in risk (4%) with an increase in consumption of 200 g/day of total dairy food. For 200 g/day of milk, the observed decrease was 2% and did not reach statistical significance. A meta-analysis of 22 prospective cohort studies and five case-control studies [6] concluded that dairy consumption was inversely associated with the risk of developing breast cancer. Dairy intakes in the reviewed studies were converted from servings or other units into grams/day. Comparison of high and moderate consumption (>600 g/day and 400–600 g/day) relative to low total dairy product consumption (<400 g/day) showed a significant decrease in breast cancer risk of 10% for high and 6% for modest total consumption, respectively, relative to low total consumption. Further linear trend and subgroup analysis showed that effect was dependent on dose and dairy type. Another meta-analysis, specifically focusing on evaluating low-fat/skim milk, whole milk and yogurt [7], based on studies from the US, Europe and Japan, observed summary OR’s suggesting a decrease in risk for high vs. low consumption of low-fat/skim milk of 14%, whole milk of 5% and yogurt of 10%. However, none of these observed decreases reached statistical significance. The evaluation of the effect of dairy products grouped by some characteristic, individually, or total consumption, provided inconsistent results. Therefore, a recent study attempted to link dairy product consumption and breast cancer risk by conducting dietary pattern analysis. Dairy products belonged to two derived dietary patterns. Evaluation for association with the risk of breast cancer failed to show a significant role for the patterns containing dairy products considered in the study [8]. Two recent reports from cohort studies observed conflicting results. A study by Fraser [9], based on the Adventist Health Study-2, observed an increase in breast cancer risk with increased milk consumption, while results from the SUN Cohort in Spain [10] observed a significant inverse association for women with moderate total dairy and moderate low-fat dairy product consumption relative to their respective low categories. In the stratified analysis by menopausal status, the inverse association of moderate total dairy product consumption was significant for postmenopausal breast cancer, and the inverse association with intermediate low-fat dairy products was significant for premenopausal breast cancer.

This lack of consistency regarding the role of dairy foods as a group (and milk) in the etiology of breast cancer was already noted in previous collective Breast Cancer Reports [11]. In the recently updated Report (2), after summarizing the results of many studies, the CUP (Continuous Update Project) expert panel concluded that with the current state of knowledge, only limited-suggestive evidence exists for a decrease in breast cancer risk with higher consumption of dairy products for premenopausal breast cancer. For diets high in calcium content, although a consistent significant protective relationship was observed for both premenopausal and postmenopausal women, the panel concluded that due to the small number of studies, only limited evidence existed [12]. The conclusions of the latest publication (4) linked effects of fermented and low-fat dairy with different breast cancer subtypes and menopausal status and also discussed the potential limitations of meta-analyses resulting from the methodological differences and emphasized the need for further well-defined studies.

This means that the problem of assessing the relationship between dairy products and breast cancer risk remains open. Milk and dairy products are too important a food group in the diet of many societies to leave the conclusion about their role in breast cancer risk undefined. Additional studies evaluating the association between dairy product consumption and breast cancer risk are needed.

The ambiguous results of the meta-analysis carried out so far, and the lack of strong evidence regarding the relationship of dairy foods with breast cancer risk in the updated Reports [1,2,7,11] prompted us to assess the relationship between milk and dairy products and breast cancer risk in a case-control study of Polish women. Considering the complexity of the nutritional research, namely the fact that the nutritional factors have a long-term effect and precede cancer development for many years, our study analyzed the habitual consumption of milk and dairy products 10–15 years prior to breast cancer diagnosis.

The aim of this study is to evaluate the relationship between the usual milk and dairy products consumption from 10 to 15 years before breast cancer diagnosis, based on data collected in an epidemiological case-control study of breast cancer in Polish women in three different regions of Poland.

## 2. Methods

### 2.1. Study Overview

This case-control study is the Polish component of the two-arm population-based case-control study (Polish native women in Poland and Polish migrant women residing in Cook County, Il and Detroit Metropolitan Area, MI, USA) entitled: “Breast Cancer in Women of Polish Ancestry” funded by the National Cancer Institute, National Institutes of Health, USA. The study is known in the field as “The Polish Women’s Health Study (PWHS)” and was initiated in the years 2001–2003. A review of the literature on breast cancer incidence in Poland showed that in the 1980s, the incidence of breast cancer in Poland was much lower compared to other European countries or US. The goal of the Polish arm of the PWHS was to evaluate the effect of the traditional Polish diet on breast cancer risk. Therefore, the time period of 1985–1989 was chosen for the assessment of dietary and other exposures because the year 1989 brought, in Poland, changes from centrally planned to a free-market economy that influenced social and economic changes, including changes in dietary habits.

### 2.2. Study Population

Breast cancer cases were identified in several Regional Cancer Registries from three regions of the country: western Poland in Poznań, the Wielkopolskie Cancer Center and the Center for Prevention and Epidemiology of Cancer; southern Poland in Gliwice at the Oncology Center and in Katowice at the Department of Internal Diseases and Oncological Chemotherapy of the Silesian Medical Academy; and eastern Poland in Białystok, the Białystok Oncology Center. The recruitment of incident breast cancer cases aged 20–79 years old from each registry started simultaneously with cases diagnosed on 1 January 2000 and continued until 200 consecutively diagnosed cases were identified. Cases were not eligible to participate if they had a previous diagnosis of in situ or invasive breast cancer or any other cancer except common skin cancer. Population-based controls were frequently matched to the age and residence distribution of breast cancer cases in 1999 at each of the five involved Oncological Centers. Women for the control sample were drawn from the national population registry (PESEL) kept by the relevant government institutions for every center separately. The selection process of controls ensured that a random sample of potential controls was chosen to reflect the frequency distribution based on 5-year age groups and place of residence of the breast cancer cases. Potential controls who, at initial screening, disclosed that they were diagnosed with breast cancer or any other cancer, except common skin cancer, before 1 January 2000, were excluded from the control group. The total number of participants in the study was 1734. After quality verification of each questionnaire, 35 questionnaires were rejected due to overestimated energy consumption, a previous diagnosis of breast or other cancers among controls not disclosed at initial screening, previous diagnosis of breast or other cancers prior to 1 January 2000, among cases, and exclusion of participants who were 80 years or older at the time of the interview. Therefore, the final sample included 1699 women aged 26–79 years of age, of which 823 are breast cancer cases and 876 population-based controls, frequency matched on age in 5-year age interval and place of residence to the cases. The response rates for the cases were 72.4% and for controls 50.7%. The study was approved by the Bioethics Committees in Poland and the USA.

### 2.3. Exposure Assessment

#### 2.3.1. Dietary Habits

Usual dietary intake during the 1985–1989 time period used a validated, semiquantitative food frequency questionnaire (FFQ) modeled on the Nurses’ Health Study (NHS) 1986 long questionnaire and supplemented with traditional Polish products and dishes. The questionnaire consisted of 130 questions about food items. The frequency of consumption was assessed in times per day, week, month, or year. For bread, eggs, bacon and alcoholic beverages, the typical serving size was ascertained. Otherwise, the average serving size was assumed.

FFQ section entitled “Dairy Foods” included questions about usual consumption of (1) cow’s milk: low fat, skimmed and whole milk (including milk in milk soups and on breakfast cereals), fermented milk and yogurt (yogurt was grouped with milk because over 80% of women never consumed yogurt during 1985–1989 since it was just being introduced to the market); (2) cottage cheese: including farmer’s cheese and homogenized cheese; (3) hard cheese: including yellow cheeses, Camembert and Brie style cheeses and other processed cheeses. Average serving sizes for these three dairy groups were assumed to be: 220 mL milk, 60 g cottage cheese and 30 g hard cheese.

In order to help participants with the recall of their usual diet during that time period, thus reducing recall bias, two graphic aids were developed and used. First, a life event calendar was introduced at the start of the interview, which allowed women to enter important life events such as marriages, births, change in residence or other important events that occurred during 1985–1989. The date of the Chernobyl accident, 26 April 1986 (4/26/1986), was also written into the calendar. This helped participants to place themselves from the start of the interview into the time period for which dietary and other lifestyle exposures were collected in the questionnaire. Second, for dietary assessment, a set of graphic cards with names of the foods and dishes corresponding to those in each question within a section was shown to the participants to help them recall the frequency of consumption for these products. Thus, when the question about the consumption of specific food was read by an interviewer, participants not only heard the name of the food but also were able to see it written as well as see a picture for some products/foods. Interviewers were instructed and had prompts in the questionnaire to frequently remind participants about the time period for which the participant was to report her food consumption. The FFQ interview was performed in the same way in the case and control sample, under as similar a condition as possible. FFQ concerned the years 1985–1989, i.e., period of 10–15 years before clinical diagnosis (in 2000) for cases and year of interview for healthy controls. The interviewers were trained nutritionists, doctors or nurses.

#### 2.3.2. Other Exposures/Covariates

For several exposures/covariates listed below, their occurrence was collected up to the time of the interview for controls and time of diagnosis for cases. First-degree family history was considered positive if a mother, sister or daughter had been diagnosed with BC. Age at menarche was assessed by the onset of the first menstrual period. Full-term pregnancy (FTP) was defined as a pregnancy lasting 24 or more weeks, irrespective of the outcome. Age at any first FTP was calculated from the date when the first pregnancy ended relative to the participant’s birthdate. For assessment of the use of oral contraceptive or hormone replacement therapy, participants were provided a list of hormonal preparations available during that time period for oral contraception (pills or injections) or hormone replacement therapy (pills, patches, or creams). For the following exposures/covariates, information was collected for the time period 1985–1989, thus the same time period as for dietary exposures. Body mass index (BMI = kg/m^2^) during 1985–1989 was calculated based on body weight and height reported for that time period during the interview. Physical activity included questions about all types of activities, including sleep; light, moderate and heavy household activities; recreational activities; occupational activities; and stair climbing. Total METs (Metabolic Equivalent) were calculated for each participant based on the intensity and duration of each activity during a 24-h day [13]. Total daily energy intake was calculated using average portion sizes (except for foods where portion sizes were recorded), which were obtained from the research on the diet of women in the 1980s carried out at the Department of Nutritional Epidemiology of the Institute of Food and Nutrition in Warsaw, and in consultation with the Central Statistical Office, GUS, Warsaw. By using these portion sizes, total energy intake was calculated based on tables of energy and nutrient values of products and dishes from 1985 to 1989 [14,15]. Alcohol intake during 1985–1989 included beer (bottles or cans), wine (4 oz/100 mL glass) and hard liquor (2 oz/50 mL shot). The size of each alcohol drink represented a standard serving with approximately 10 g of pure ethanol. The total number of weekly servings was calculated as the sum of the weekly servings of each type.

#### 2.3.3. Menopausal Status in 1985–1989

Given the 5-year span of the time period, we assessed the menopausal status of each participant relative to the midpoint of the time period, i.e., the year 1987. If participants were still menstruating in 1987, we assigned them to the premenopausal group. If they reported that they stopped menstruating by 1987, we assigned them to the postmenopausal group. If the information on women’s menstrual status was missing and she was over 50-years-old in 1987, we assigned her to the postmenopausal group. Classification of menopausal status corresponds to the same time period as our assessment of usual diet.

### 2.4. Statistical Methods

All analyses were performed using SAS version 9.4 (SAS Inc., Cary, NC, USA). In order to test for group differences between cases and controls, for demographic and other characteristics, we used logistic regression models adjusted for age and site: age at diagnosis (cases) or interview (controls) (<35 years; 35–44 years; 45–54 years; 55–64 years; 65–74 years; ≥75 years) and site (Gliwice, Katowice, Poznań WCO, Poznań OPEN, Białystok). To test for case/control differences between the distribution of total and individual dairy products consumption as continuous variables, due to skewness of these distributions, a nonparametric equivalent of a two-sample *t*-test, the Mann–Whitney test, was used. Similarly, to evaluate the correlation between individual dairy products consumption, a Spearman correlation coefficient was calculated.

To evaluate the association between total and individual dairy products consumption and risk of breast cancer, stratified by menopausal status in 1987, we used multivariable logistic regression to estimate adjusted odds ratios (ORs) and 95% confidence intervals (95% CI). To evaluate these associations, we categorized consumption in servings/week as follows: total (0–≤3.5, 3.5–≤7, 7–≤10.5, 10.5–≤14, 14–≤17.5, 17.5–≤21 and >21) and milk consumption in glasses/week (0–≤3.5, 3.5–≤7, 7–≤10.5, 10.5–≤14 and >14). For cottage cheese, we used terciles of servings/week (0–≤1.5, 1.5–≤2.5, >2.5), and for hard cheese, we used quartiles of servings/week (0–<1.5, 1.5–<3, 3–<4.5, ≥4.5) among controls, respectively. Because servings/week cluster at certain values, the distribution of controls for terciles was the closest serving size to 33% and 25% for quartiles. Two participants were missing information for the consumption of cottage cheese, and one for hard cheese. They were assigned median value specific for the site, age group and case/control status.

Model 1 assessed the association, measured by OR and 95% CI, between breast cancer risk and increasing consumption as defined by the specific categories for total and each dairy product, relative to the respective low category of consumption. In Model 1, we adjusted only for the two variables on which the cases and controls were frequency matched by design, site and age categories. In Model 2, we additionally adjusted for all potential confounders, and in Model 3, we also included all three dairy products to obtain mutually adjusted (for the consumption of the other two dairy products) ORs and 95% CI. The ORs from Model 3 represents the independent effect of a given dairy product after adjustment for consumption of the other two dairy products, accounting for co-exposure and collinearity between consumption of these products.

The potential confounders included in Model 1 are: age and site: age at diagnosis (cases) or interview (controls) (<35 years; 35–44 years; 45–54 years; 55–64 years; 65–74 years; ≥75 years) and site (Gliwice, Katowice, Poznań WCO, Poznań OPEN, Białystok). In Model 2, we additionally adjusted for: first degree family history of breast cancer (yes/no); age at menarche (<13, 13–<14, 14–<15, ≥15); age at first full-term pregnancy (nulliparous, <22, 22–<30, ≥30); use of OC (yes/no); use of hormone replacement therapy (yes/no); BMI in 1895–1989 (<18.5, 18.5–<25, 15–<30, ≥30); physical activity in 1985–1989 in quartiles of METs/day for controls (≤1.96, 1.96≤ 2.2, 2.2–≤2.5, >2.5); total energy intake (kcal/day) in quartiles for controls (<1598, 1598–<1924, 1924–<2344, ≥2344); alcohol intake (drinks/week) in 1985–1989 (none, <0.5, ≥0.5). For Model 3, we added all individual dairy products to Model 2.

To test for linear trend, which assessed for dose–response relationship for total and each of the dairy products (serving/week), we used median values for each of the categories of consumption and entered it as a continuous variable in the model. This provides us with OR and 95% CI for the trend of one serving/week for total consumption and consumption of a given dairy product. Models for the linear trend are adjusted for the same potential confounders as assessment of ORs for the individual categories of consumption in Model 1, Model 2 and Model 3. A *p*-value <0.05 was considered statistically significant, “ns” represents a non-significant *p*-value.

## 3. Results

The distribution of selected characteristics by case/control status is presented in Table 1. A significantly higher proportion of cases reported having a family history of a first-degree relative with breast cancer, a lower proportion had a later age at menarche, a higher proportion had late age at first full-term pregnancy and a higher proportion was classified as having higher BMI ≥ 25. Although by design, cases and controls were frequency matched on 5-year age intervals, we observed a significantly higher proportion of controls 65 years and older. Age was always included as an adjustment variable in analyses. Cases were similar to controls in their distribution within site, use of oral contraception, use of hormone replacement therapy, physical activity, total energy intake and alcohol intake.

Table 2 provides percentile distribution (25th, 50th and 75th) for total dairy products consumption as well as individual dairy products, for cases and controls by menopausal status, and the *p*-values from the Mann–Whitney test, which compared the distributions of consumption between cases and controls. For total dairy products consumption, continuous distribution of servings/week did not differ significantly between cases and controls for either menopausal status. For milk, the controls had significantly higher consumption than cases in both groups of menopausal status. For cottage cheese, a higher consumption by controls was observed only for postmenopausal women and for hard cheese, a reverse was observed, a higher consumption by cases and only in premenopausal women.

We also evaluated the pattern of co-consumption by participants of the three dairy groups (milk, cottage cheese and hard cheeses), using Spearman correlation analysis (Table 3). This analysis showed us the degree of co-consumption and collinearity between these products. We observed consistent significant positive correlation across the four subgroups, premenopausal case/control, postmenopausal case/control, between milk consumption and cottage cheese, ranging between 0.24 and 0.33, and between the two types of cheeses, ranging between 0.24 and 0.35; all were statistically significant at *p* < 0.001. The correlation between milk consumption and hard cheeses was not significant for premenopausal controls and postmenopausal cases, positive for premenopausal cases (r = 0.12, *p* = 0.003) and negative for postmenopausal controls (r = −0.14, *p* = 0.028)

We evaluated the association between dairy product consumption and risk of breast cancer, separately within the strata of participants’ menopausal status in 1987 (Table 4). Odds ratios (ORs) and 95% CI reported for Model 1 represent the risk of being a case (decreased or increased) for a given consumption category of total dairy products as well as the individual dairy product, relative to the reference category, adjusted for the two variables on which cases and controls were frequency matched (site and age categories). For Model 2, the reported ORs represent the risk of being a case (decreased or increased) additionally adjusted for all the potential confounders. For Model 3, the reported ORs represent the risk of being a case (decreased or increased) additionally adjusted for the consumption of the other two dairy products, thus representing the independent effect of the given product on breast cancer risk, when adjusted for the mean consumption of the other two products. Thus, mutual adjustment of multiple exposures impacts the observed independent association effects due to co-exposure and collinearity of exposures (mutually adjusted ORs not shown in Table 4 but provided and discussed in the text below).

The multivariate adjustment for potential confounders (Model 2) did not change our OR estimates substantially from those observed in Model 1, with the exception of total dairy consumption for premenopausal women. In Model 1, a statistically non-significant OR _trend_ = 0.99, *p* = 0.24 was observed, while after adjustment for the potential confounders (Model 2), it reached statistical significance, OR _trend_ = 0.98, *p* = 0.01. For Model 2, premenopausal women (for milk), we observed a significant decrease in risk of breast cancer with increasing milk consumption. For the highest category of consumption (>14 glasses/week, or >2 glasses/day), relative to ≤3.5 glasses/week, or half a glass per day, we observed a 56% reduction in breast cancer risk, which reached statistical significance (OR = 0.44, 95% CI 0.29–0.67). The model for linear trend, using medians for each category, showed a significant decrease in risk (OR _trend_ = 0.95, *p* = 0.0002), i.e., 5% decrease in risk of breast cancer for an increase in milk consumption of one glass/week. The consumption of cottage cheese was not associated with the risk of developing breast cancer for premenopausal women. The consumption of hard cheese showed a significant increase in risk for consumption of >3–<4.5 servings/week with OR = 1.60, 95% CI = 1.16–2.22. The consumption of ≥4.5 servings/week also increased risk, however, it did not reach our defined statistical significance of *p* < 0.05 (OR = 1.47, 95% CI = 1.00–2.17, *p* = 0.0517). The model for linear trend across the medians for each consumption category of hard cheese, evaluated as a continuous variable, showed a significant increase in risk with an increase in consumption of one serving/week (OR _trend_ = 1.10, 95% CI = 1.02–1.18, *p* = 0.015) or 10% increase in the risk of developing breast cancer, with an increase in consumption of one serving/week of hard cheese.

For postmenopausal women (Model 2), total dairy products consumption showed a significant decrease for one category (>17.5–≤21) servings/week (OR = 0.23, CI = 0.09–0.63). The linear trend, although of similar magnitude as for premenopausal women, did not reach statistical significance, potentially due to the smaller sample size (OR _trend_ = 0.98, 95% CI = 0.94–1.01, *p* = 0.20). For milk, we observed a significant decrease in risk for breast cancer of 59% (OR = 0.41, 95% CI = 0.19–0.88) for women who were consumers of (10.5–≤14) glasses of milk/week, relative to low consumers. For women who were in the next higher category of milk consumption (>14 glasses/week), relative to low consumers, a decrease in risk was also observed (OR = 0.55), which did not reach statistical significance (95% CI = 0.27–1.13). The linear trend for a decrease in risk with increasing consumption of milk in postmenopausal women was of the same magnitude as for premenopausal women and was statistically significant (OR _trend_ = 0.95, *p* = 0.03), i.e., 5% decrease in risk for an increase in consumption of one glass/week. Increasing consumption of cottage cheese, in contrast to premenopausal women, showed a significant protective effect. The ORs for the two categories (>1.5–≤2.5, and >2.5 servings/week), relative to reference category (≤1.5 servings/week), are OR = 0.57, 95% CI = 0.33–0.99 and OR = 0.48, 95% CI = 0.29–0.80, respectively. The linear trend for decreased risk is statistically significant with OR _trend_ =0.80, *p* = 0.009, or a 20% reduction in risk with an increase in consumption of cottage cheese by one serving/week. Consumption of hard cheese did not show statistically significant increases across the categories; however, for the highest category of consumption, ≥4.5 servings/week, a non-significant increase in risk was observed (OR = 1.81, 95% CI = 0.89–3.65). A linear trend was of the same order of magnitude as for premenopausal women; however, it did not reach statistical significance potentially due to the smaller sample size (OR _trend_ = 1.11, *p* = 0.15), i.e., 11% increase in risk for an increase in consumption of one serving/week of hard cheese.

Additionally, we ran Model 3, which included all potential confounders from Model 2, as well as all three dairy products as co-exposures. For premenopausal women, the observed pattern remained similar to that observed for individual products in Model 2. A significant reduction in risk was observed with increasing intake of milk (OR _trend_ = 0.95, *p* = 0.0002), no association with consumption of cottage cheese and an increase in risk for hard cheese (OR _trend_ = 1.09, *p* = 0.04). For postmenopausal women, the pattern for cottage cheese remained the same as in Model 2, i.e., a significant decrease in risk for an increase in consumption of cottage cheese of one serving/week (OR _ltrend_ = 0.79, *p* < 0.01). However, due to the high correlation between milk and cottage cheese consumption, the independent effect of milk consumption measured by adjusted OR for the decrease in risk with increased milk consumption did not reach statistical significance (OR _trend_ = 0.97, *p* = 0.13). The pattern observed for hard cheese in the mutually adjusted Model 3 was similar to that observed in Model 2 and also did not reach statistical significance, although the point estimate was of similar magnitude as in premenopausal women (OR _trend_ = 1.15, 95% CI = 0.99–1.33, *p* = 0.063). However, a significant increase in risk was observed for the highest category (≥4.5 servings/week) of hard cheese consumption (after adjustment for consumption of the other two dairy products). The observed OR for the highest category relative to the reference category was: (OR = 2.21, 95% CI = 1.04–4.73).

## 4. Discussion

Motivated by the inconsistencies of findings for total dairy product consumption in relation to breast cancer risk, in addition to analysis of total dairy consumption, we also chose to analyze each dairy group (milk, cottage cheeses and hard cheeses) separately.

Our findings confirmed that total dairy product consumption does not capture the bidirectional relationship of individual dairy groups with the risk of breast cancer. We found in the adjusted model for potential confounders (Model 2) that for premenopausal women, although a significant decrease in risk was observed for an increase in one serving/week of total dairy products (OR _trend_ = 0.98, *p* = 0.01), this decrease was of a smaller magnitude than the decrease observed for milk alone (OR _trend_ = 0.95, *p* = 0.0002). Moreover, for postmenopausal women, no decrease in risk was observed for total dairy consumption. However, high consumption of milk decreased breast cancer risk for both premenopausal and postmenopausal women. For both groups, the magnitude of reduction per one glass/week of milk consumption, evaluated by a linear trend, was the same (5% reduction, OR _trend_ = 0.95), and both estimates reached statistical significance. A reverse relationship was observed for hard cheeses. For premenopausal women, a statistically significant increase in risk (10% increase OR _trend_ = 1.10) was observed for each increase in consumption of one serving/week. For postmenopausal women, the pattern of increase in risk was not consistent, although the linear trend for an increase in risk was similar in magnitude (OR _linear trend_ = 1.11); however, it did not reach statistical significance. Potential reasons could be the smaller sample size for postmenopausal women and inconsistency in the observed pattern. Cottage cheeses, which are closer in their content of calcium and other nutrients to milk than hard cheeses, also had a different relationship with risk by menopausal status. There was no significant association with risk for premenopausal women and strong protective effect for postmenopausal women. The linear trend for postmenopausal women was OR _trend_ = 0.80 and was highly statistically significant with a value of *p* = 0.009. Our findings illustrate the need for a separate analysis of each dairy product group because of their bidirectional effect on breast cancer risk, as well as stratified by menopausal status.

To evaluate patterns of co-consumption of these dairy groups, we carried out a correlation analysis. We observed a high positive correlation between consumption of milk and cottage cheese and between the two types of cheeses for all four groups classified by menopausal and case/control status. However, the correlation between milk consumption and hard cheeses was inconsistent; either null, low positive or low negative. The observed pattern of correlations suggests that women who consumed milk also consumed cottage cheeses but did not consume as many hard cheeses, supporting the existence of different patterns of habitual consumption related to dairy products and further pointing to the need for separate analyses for each of these dairy groups.

Our analysis carried out in Model 3, where consumption of the individual dairy products was mutually adjusted for the other two groups, reflects the impact of co-consumption on the estimate of the independent effects of a given group when consumption of other groups is accounted for in the model. Although the pattern of protection of decrease or increase in risk was similar to that observed for Model 2, for postmenopausal women, due to a significant positive correlation between milk consumption and cottage cheese, the previously observed reduction in breast cancer risk with increasing consumption of milk did not reach statistical significance after the strong protective effect of cottage cheese was accounted for in the model.

In the literature, specifically for milk, there are hypotheses for both the protective as well as increased risk of breast cancer due to increased milk consumption, which are based on nutrients and other components potentially present in milk. Our result joins the previous articles in which increased consumption of milk was observed to reduce breast cancer risk in women from Japan [16], Finland [17], China [18] or the USA [19] and in the Canadian Breast Screening Study [20].

For the other two product groups, cottage cheeses and hard cheeses, little research has been published on their individual association with breast cancer. In most studies, they are included in the general category of dairy products or, as in pooled analysis, they appear together as “total dairy solids,” sometimes with butter included [20]. In the few reports on the total consumption of cheeses, without the division into cottage cheese and hard cheeses, their role in the etiology of breast cancer has not been consistent [9,17,19,21,22].

Many studies analyze milk and other dairy products as total dairy products consumption and their relationship with breast cancer risk. In our study, total dairy consumption did not reflect the magnitude of protection that was observed for milk. This group is not only the main source of calcium in the diet but also many other macro and micronutrients and many biologically active components, which can either protect against or increase breast cancer risk. The nutrients with the potential to reduce breast cancer risk are calcium, vitamin D, conjugated linoleic acid (CLA), butyric acid and vaccenic acid, as well as whey proteins and the composition of microorganisms in the fermented products.

The presence of calcium and its homeostasis regulate the balance in blood serum and tissues. Calcium promotes cell differentiation, reduces proliferation and enhances apoptosis. The functioning of calcium is modulated by vitamin D, which also induces apoptosis, reduces cell proliferation and promotes the ability to differentiate cells [2,23,24,25].

Linoleic acid with CLA coupled bonds may affect the development of cancer both by directly interfering with the carcinogenesis process and indirectly by reducing the amount of adipose tissue in the body. Of particular importance is the action of CLA isomers in the neoplastic process within the mammary glands at various stages: initiation, promotion and progression [26,27]. Studies of vaccenic acid (TVA, trans-11 C18: 1), which is an oleic acid isomer and the main trans isomer in ruminant fats [26], suggest a linear increase in CLA synthesis with increasing vaccenic acid consumption [28]. Vaccenic acid is present in large amounts in food products, including milk derived from polygastric animals. The conversion of vaccenic acid to cis-9, trans-11 CLA, results in an increased accumulation of this compound in the adipose tissue of the mammary glands and their lower susceptibility to chemically induced carcinogenesis. The inhibitory effect of the neoplastic process was already observed when a 1% addition of vaccenic acid was used in the animals’ diet [29,30,31,32]. Butyric acid, which is present in lactic fat, also exhibits strong anti-cancer properties because it induces cell differentiation and apoptosis and inhibits cell proliferation and angiogenesis in many cell lines, including breast cancer [24,33,34,35].

Studies that come to the conclusion that increased milk consumption increases breast cancer risk include pooled analysis [20], cohort studies in Norway [36,37] and the USA [38] and in the Adventist Health Study (9), as well as in the meta analyzes developed by Dong (5) and Chen (7). The observed increase in risk is hypothesized to be due to the presence of estrogens, insulin-like growth factor (IGF-1) and saturated fatty acids in milk and its products. Milk contains small amounts of steroid hormones, including estrogens such as 17 ß estradiol (E2), estrone (E1) and estriol (E3) [39,40]. The level of estrogen in cow’s milk depends on the physiological condition of cows and increases during pregnancy. The amount of estrogen consumed with dairy products is believed to be negligible, and other authors suggest that it is too small to increase the risk of breast cancer development [41,42]. In contrast, the concentration of IGF-1 may be higher than that of estrogen in cow’s milk, since in some countries, cows are administered synthetic growth hormone (somatotropin-rbST) [43] in order to increase the yield of milk. Since IGF-1 stimulates cell growth and differentiation and inhibits apoptosis, the presence of this hormone in milk has the potential to influence the development of cancerous tumors [44] and the development of breast cancer [45,46]. In many studies, saturated fatty acids were thought to increase the risk of breast cancer [47,48,49]. However, other studies indicate that it is rather the high ratio of n-6/n-3 fatty acids that might be responsible for the increase in breast cancer risk [50,51,52,53].

The contents of the many ingredients in milk and dairy products that can potentially reduce or increase breast cancer risk are not equivalent when measured in 100 g of the product or in terms of usual serving size consumed as shown in Food Composition Tables. A recent publication [54] for the selected components such as calcium and phosphorus provides an example of the different contents of these nutrients and their ratio Ca:P in the different dairy products. One should also consider milk sugar, lactose, which is also present in different quantities in individual dairy products, which increases the bioavailability of calcium by passive diffusion in the intestinal villi [55].

The different dietary habits with respect to consumption of dairy products in different countries, thus affecting the distribution of the proportion of consumed individual products, the lack of equivalency for the content of the nutrients and other substances, hypothesized to increase or decrease breast cancer risk, and our findings for the different effect of individual dairy group products, point out that dairy products composition is heterogenous and each product groups should be analyzed individually.

Finally, it is worth considering that the collection of information on dietary habits, the Food Frequency Questionnaire (FFQ), which is the method of choice for studying the effect of nutritional habits and cancer risk [56], could also be contributing to the observed heterogeneity of findings. In most case-control studies, that information is collected for a year prior to diagnosis for cases and reference date (usually one year before the interview) for controls. However, diet and the nutrients that we consume are important at every stage of carcinogenesis and can impact the initiation, promotion as well as the progression of cancer [57]. Therefore, our analyses of dairy consumption from 10 to 15 years prior to cancer diagnosis brings in a prospective component of adult diet to our analysis and thus assesses the role that these products could play in the full process of breast cancer carcinogenesis.

The strengths of our study include: the standardized in-person interview of cases and controls by highly trained personnel, nutritionists, physicians and nurses, who were aware of the potential for recall and information bias if administration of the questionnaire and probing for answers was not consistent between cases and controls; use of FFQ, the method of choice for collecting information on usual diet from any time period, both in case-control as well as cohort studies evaluating the association of dietary products with cancer risk; the well-defined time period of reference, memorable as it immediately preceded major economic and political changes in Poland; use of life events calendar to help participants bring back the memories of that time period and use of graphic show cards to allow for better understanding of a question about given product/food by allowing participants to read the question and not just hear it; collection of relevant potential confounders, such a physical activity and weight, for the same time period as the assessment of the diet and our ability to classify participants on their menopausal status during that time period; and finally as mentioned above, analysis of dairy products consumption 10–15 years prior to diagnosis, which allows us to evaluate their role in the process of carcinogenesis that occurs over a long time period, when habitual intake of dairy products and their interaction with women’s hormonal status can act either as anti-promoters or promoters in the process of carcinogenesis.

There are also several limitations. First, our sample size was limited, especially for postmenopausal women. Therefore, some of the estimates for linear trends in postmenopausal women, similar in magnitude to those of premenopausal women, did not reach statistical significance at α = 0.05. Similarly, due to limited sample size, our power was limited to assess statistically for the heterogeneity of associations (by including interaction terms in our models) for menopausal status as well as other potential modifiers such as family history or BMI. Second, as in any case-control study, diet recall was retrospective, and thus the potential for recall bias cannot be excluded. Although our referent time period was 10–15 years before the interview, women could identify with important events such as the Solidarity movement in Poland or the 1986 Chernobyl atomic reactor accident, thus more easily recalling their usual diet at that time. Given that the literature is inconsistent about the effect of dairy products on breast cancer risk, we do not believe that recall/information bias would be differential by case/control status; of course, we are not able to test this assumption. Finally, even though we adjusted for many reproductive and other lifestyle factors for breast cancer, residual confounding cannot be excluded.

## 5. Conclusions

In summary, we conclude that the results of our study, which includes analysis of both the total dairy products consumption as well as analysis by individual dairy products, strongly supports the view that analysis of total consumption does not reflect the bi-directional relationship of specific dairy products with the risk of developing breast cancer. In our study, milk consumption is associated with a reduced risk of breast cancer in both premenopausal and postmenopausal women, while the protective effect of cottage cheese achieved statistical significance only in postmenopausal women. In contrast, a significant increase in breast cancer risk was observed with increases in the consumption of hard cheeses in premenopausal women. Total consumption of dairy products showed a significant reduction in risk only for premenopausal women. The magnitude of risk reduction was smaller than that observed for milk, the largest component of total dairy consumption in our sample. Given the bi-directional effect of the individual dairy products on breast cancer risk, the magnitude and direction of the association between total dairy product consumption and breast cancer risk will be influenced by the culturally determined distribution of the individual dairy products consumption in any given population. Thus, further research into the effect of dairy products consumption in relation to the preclinical stages of breast cancer development is desirable because of their role in the usual diet of populations and their potential role in breast cancer prevention.

## Figures and Tables

**Table 1:** Selected characteristics of breast cancer cases and controls residing in Gliwice, Katowice, Poznań and Białystok.

Variable	Cases	Controls	*p*-Value ^1^
Sample Size	(*n* = 823)	(*n* = 876)	
	*n* (%)	*n* (%)	
Site			ns ^2^
Gliwice	200 (24.3)	201 (23.0)	
Katowice	198 (24.0)	208 (23.7)	
Poznań WCO	147 (17.9)	160 (18.3)	
Poznań OPEN	101 (12.3)	113 (12.9)	
Białystok	177 (21.5)	194 (22.1)	
Age (years) at diagnosis-cases/interview-controls			0.001 ^3^
<35	17 (2.1)	33 (3.8)	
35–44	124 (15.1)	108 (12.3)	
45–54	303 (36.8)	268 (30.6)	
55–64	207 (25.2)	197 (22.5)	
65–74	136 (16.5)	212 (24.2)	
≥75	36 (4.4)	58 (6.6)	
First-degree family history of breast cancer	76 (9.2)	46 (5.3)	0.001
Age at menarche (years)			0.03
<13	228 (27.7)	227 (25.9)	
13–<14	194 (23.6)	169 (19.3)	
14–<15	226 (27.5)	236 (26.9)	
≥15	175 (21.3)	244 (27.9)	
Age at first full-term pregnancy (years)			0.001
Nulliparous	84 (10.2)	82 (9.4)	
<22	214 (26.0)	247 (28.2)	
22–<30	438 (53.2)	497 (56.7)	
≥30	87 (10.6)	50 (5.7)	
Ever used oral contraception	90 (10.9)	83 (9.5)	ns
Ever used hormonal replacement therapy	130 (15.8)	117 (13.4)	ns
BMI in 1985–1989 (kg/m^2^)			0.02
<18.5	42 (5.1)	34 (3.9)	
18.5–<25	462 (56.1)	507 (57.9)	
25–<30	232 (28.2)	218 (24.9)	
≥30	60 (7.3)	58 (6.6)	
Missing	27 (3.3)	59 (6.7)	
Physical activity in 1985–1989 (MET) (quartiles)			ns
I (≤1.96)	193 (23.5)	218 (24.9)	
II (1.96–≤2.2)	201 (24.2)	220 (25.1)	
III (2.2–≤2.25)	186 (22.6)	219 (25.0)	
IV (>2.25)	243 (29.5)	219 (25.0)	
Total energy intake in 1985–1989 (kcal/d) (quartiles)			ns
<1598	199 (24.2)	219 (25.0)	
1598–<1924	190 (23.1)	220 (25.1)	
1924–<2344	218 (26.5)	219 (25.0)	
≥2344	216 (26.3)	218 (24.9)	
Alcohol intake in 1985–1989 (drinks/week)			ns
none	120 (14.6)	132 (15.1)	
<0.5	476 (57.8)	546 (62.4)	
≥0.5	227 (27.6)	197 (22.5)	
Menopausal states in 1985–1989			0.001
premenopausal	610 (74.1)	619 (70.7)	
postmenopausal	213 (25.9)	257 (29.3)	

^1^ Comparison between cases and controls, adjusted for age at diagnosis (cases) or interview (controls) (<35 years; 35–44 years; 45–54 years; 55–64 years; 65–74 years; ≥75 years) and site (Gliwice, Katowice, Poznań WCO, Poznań OPEN, Białystok). ^2^ Adjusted for age at diagnosis (cases) or interview (controls). ^3^ Adjusted for site.

**Table 2:** Percentile distribution, for dairy products consumption (servings/week), for premenopausal and postmenopausal women.

Dairy Products	Percentiles *p*-Value *	Premenopausal		Postmenopausal	
		Controls (*n* = 619)	Cases (*n* = 610)	Controls (*n* = 257)	Cases (*n* = 213)
Total dairy	75th	18.63	17	19.8	18.73
	50th	11.34	11	13.5	11.86
	25th	6.84	6.46	8.46	6.8
	*p*-value		(ns)		(ns)
Milk	75th	11.8	10.2	14	12.6
	50th	7	5.8	8	7
	25th	2.78	2	3.8	2.88
	*p*-value		**		**
Cottage cheese	75th	3	3	3	3
	50th	2	2	2	2
	25th	1	1	1.15	1
	*p*-value		(ns)		**
Hard cheese	75th	3	4	3	3
	50th	2	2	2	2
	25th	1	1.23	1	0.92
	*p*-value		**		(ns)

* *p*-value from Mann–Whitney U test. ** Distribution of consumption differs at *p* < 0.05 between cases and controls.

**Table 3:** Dairy products correlations (Spearman r) and corresponding *p*-values among Polish women residing in Gliwice, Katowice, Poznań and Białystok.

Spearman r (*p*-Value)				
Premenopausal				
	Controls (*n* = 619)		Cases (*n* = 610)	
Dairy Product	Milk	Cottage Cheese	Milk	Cottage Cheese
Milk	1.0 *		1.0	
Cottage Cheese	0.32 (<0.001)	1.0	0.28 (<0.001)	1.0
Hard Cheese	0.06 (ns)	0.28 (<0.001)	0.12 (0.003)	0.35 (<0.001)
Postmenopausal				
	Controls (*n* = 257)		Cases (*n* = 213)	
Dairy Product	Milk	Cottage Cheese	Milk	Cottage Cheese
Milk	1.0		1.0	
Cottage Cheese	0.24 (<0.001)	1.0	0.33 (<0.001)	1.0
Hard Cheese	−0.14 (0.028)	0.26 (<0.001)	0.04 (ns)	0.24 (<0.001)

* r = 1, represents correlation of a given product with itself, *p*-value not applicable.

**Table 4:** Odds ratios (ORs) and 95% confidence interval (95% CI) of breast cancer by the dairy products consumption among Polish women residing in Gliwice, Katowice, Poznań and Białystok.

Dairy Products	Case *n* (%)	Control *n* (%)	Model 1 ^a^			Model 2 ^b^		
			OR	95% CI	Trend OR (95% CI) *p*-Value	OR	95% CI	Trend OR (95% CI) *p*-Value
**Premenopausal Women (610 Cases, 619 Controls)**								
**Total Dairy Products**								
0–≤3.5	55 (9.0)	54 (8.7)	1.00 (referent)		0.99 (0.98–1.01) ns	1.00 (referent)		0.98 (0.96–0.99) 0.01
>3.5–≤7	117 (19.2)	109 (17.6)	1.14	0.72–1.81		1.12	0.69–1.80	
≤10.5	126 (20.7)	112 (18.1)	1.20	0.76–1.90		1.09	0.70–1.77	
>10.5–≤14	91 (14.9)	115 (18.6)	0.83	0.52–1.34		0.75	0.46–1.23	
>14–≤17.5	81 (13.3)	59 (9.5)	1.46	0.88–2.43		1.14	0.66–1.99	
>17.5–≤21	55 (9.0)	65 (10.5)	0.96	0.56–1.63		0.70	0.39–1.26	
>21	85 (13.9)	105 (17.0)	0.85	0.53–1.37		0.64	0.37–1.10	
Milk (glasses/week)								
0–≤3.5	233 (38.2)	208 (33.6)	1.00 (referent)		0.97 (0.95–0.99) 0.01	1.00 (referent)		0.95 (0.93–0.98) 0.0002
>3.5–≤7	123 (20.2)	122 (19.7)	0.93	0.68–1.28		0.83	0.60–1.16	
>7–≤10.5	108 (17.7)	101 (16.3)	0.96	0.69–1.35		0.80	0.55–1.14	
>10.5–≤14	64 (10.5)	64 (10.3)	0.89	0.60–1.32		0.73	0.47–1.13	
>14	82 (13.4)	124 (20.0)	0.61	0.43–0.87		0.44	0.29–0.67	
Cottage cheese (portions/week)								
0–≤1.5	218 (35.8)	237 (38.3)	1.00 (referent)		1.10 (0.96–1.25) ns	1.00 (referent)		1.06 (0.92–1.23) ns
>1.5–≤2.5	160 (26.2)	155 (25.0)	1.17	0.87–1.56		1.08	0.80–1.46	
>2.5	232 (38.0)	227, (36.7)	1.20	0.92–1.57		1.13	0.85–1.51	
Hard cheese (portions/week)								
0–<1.5	191 (31.3)	248 (40.1)	1.00 (referent)		1.12 (1.04–1.20) 0.002	1.00 (referent)		1.10 (1.02–1.18) 0.015
≥1.5–<3	152 (24.9)	164 (26.5)	1.17	0.87–1.57		1.12	0.83–1.53	
≥3–<4.5	165 (27.1)	127 (20.5)	1.66	1.22–2.27		1.60	1.16–2.22	
≥4.5	102 (16.7)	80 (12.9)	1.61	1.12–2.31		1.47	1.0–2.17	
Postmenopausal women (213 cases, 257 controls)								
Total dairy products								
0–≤3.5	21 (9.9)	16 (6.2)	1.00 (referent)			1.00 (referent)		
>3.5–≤7	37 (17.4)	33 (12.8)	0.80	0.35–1.81	0.99 (0.96–1.01) ns	0.80	0.34–1.91	0.98 (0.94–1.01) ns
>7–≤10.5	36 (16.9)	45 (17.5)	0.60	0.27–1.34		0.46	0.19–1.13	
>10.5–≤14	28 (13.1)	41 (16.0)	0.56	0.24–1.27		0.47	0.19–1.19	
>14–≤17.5	30 (14.1)	30 (11.7)	0.80	0.34–1.85		0.55	0.21–1.42	
>17.5–≤21	22 (10.3)	49 (19.1)	0.32	0.14–0.75		0.23	0.09–0.63	
>21	39 (18.3)	43 (16.7)	0.72	0.32–1.60		0.56	0.21–1.45	
Milk (glasses/week)								
0–≤3.5	64 (30.0)	62 (24.1)	1.00 (referent)		0.96 (0.93–1.0) 0.04	1.00 (referent)		0.95 (0.91–0.99) 0.03
>3.5–≤7	53 (24.9)	46 (17.9)	1.13	0.66–1.94		1.07	0.60–1.92	
>7–≤10.5	33 (15.5)	48 (18.7)	0.66	0.37–1.19		0.58	0.31–1.10	
>10.5–≤14	20 (9.4)	37 14.4)	0.56	0.29–1.08		0.41	0.19–0.88	
>14	43 (20.2)	64 (24.9)	0.66	0.38–1.14		0.55	0.27–1.13	
Cottage cheese (portions/week)								
0–≤1.5	82 (38.5)	68 (26.5)	1.00 (referent)		0.86 (0.75–0.99) 0.04	1.00 (referent)		0.80 (0.68–0.95) 0.009
>1.5–≤2.5	53 (24.9)	74 (28.8)	0.61	0.37–0.99		0.57	0.33–0.99	
>2.5	78 (36.6)	115 (44.7)	0.59	0.38–0.92		0.48	0.29–0.80	
Hard cheese (portions/week)								
0–<1.5	91 (42.7)	115 (44.7)	1.00 (referent)		1.08 (0.96–1.23) ns	1.00 (referent)		1.11 (0.97–1.27) ns
≥1.5–<3	52 (24.4)	57 (22.2)	1.17	0.72–1.89		1.16	0.69–1.95	
≥3–<4.5	39 (18.3)	60 (23.4)	0.82	0.48–1.39		0.92	0.52–1.63	
≥4.5	31 (14.6)	25, (9.7)	1.67	0.88–3.15		1.81	0.89–3.65	

^a^ Multivariable model stratified by menopausal status in 1987 and adjusted for: Age at diagnosis(cases) or interview (controls) (<35 years; 35–44 years; 45–54 years; 55–64 years; 65–74 years: ≥75 years); site (Gliwice, Katowice, Poznan WCO, Poznan OPEN, Bialystok). ^b^ Multivariable model stratified by menopausal status in 1987 and additionally adjusted for: age at menarche (<13, 13–<14, 14–<15, ≥15); age at first full-term pregnancy (Nulliparous, <22, 22–<30, ≥30); first-degree family history of breast cancer (yes, no); oral contraception use (yes, no); hormonal replacement therapy use (yes, no); BMI in 1985–1989 (<18.5, 18.5–<25, 25–<30, ≥30); physical activity in 1985–1989 in METs (1.96, 1.96–2, 2.2–2.5, >2.5); total energy intake (<1598, 1598–>1924, 1924–<2344, ≥2344); alcohol intake in 1985–1989 (none, <0.5, ≥0.05).

## Data Availability

The dataset used for the analysis of the current study is available from the corresponding author on reasonable request.
